# A longitudinal study of allergy and intestinal helminth infections in semi urban and rural areas of Flores, Indonesia (ImmunoSPIN Study)

**DOI:** 10.1186/1471-2334-11-83

**Published:** 2011-04-01

**Authors:** Firdaus Hamid, Aprilianto E Wiria, Linda J Wammes, Maria MM Kaisar, Bertrand Lell, Iwan Ariawan, Hae Won Uh, Heri Wibowo, Yenny Djuardi, Sitti Wahyuni, Robert Schot, Jaco J Verweij, Ronald van Ree, Linda May, Erliyani Sartono, Maria Yazdanbakhsh, Taniawati Supali

**Affiliations:** 1 Department of Microbiology, Faculty of Medicine, Hasanuddin University, Makassar, Indonesia; 2 Department of Parasitology, Leiden University Medical Center, Leiden, The Netherlands; 3 Department of Parasitology, Faculty of Medicine, University of Indonesia, Jakarta, Indonesia; 4 Medical Research Unit, Albert Schweitzer Hospital, Lambaréné, Gabon; Department of Parasitology, Institute of Tropical Medicine, University of Tübingen, Tübingen, Germany; 5 Department of Biostatistics, School of Public Health, University of Indonesia, Jakarta, Indonesia; 6 Department of Medical Statistics and Bioinformatics, Leiden University Medical Center, Leiden, The Netherlands; 7 Department of Parasitology, Faculty of Medicine, Hasanuddin University, Makassar, Indonesia; 8 Department of Pulmonology, Leiden University Medical Center, Leiden. The Netherlands; 9 Department of Experimental Immunology and Department of Otorhinolaryngology, Academic Medical Center, Amsterdam University, Amsterdam, The Netherlands

## Abstract

**Background:**

The prevalence of asthma and atopic disease has been reported to be low in low income countries, however helminth infections are likely to be high among these communities. The question of whether helminth infections play a role in allergic diseases can best be addressed by intervention studies. None of the studies so far have been based on a large scale placebo-controlled trial.

**Method/Design:**

This study was designed to assess how intestinal helminth infections can influence the immune response and atopic and allergic disorders in children in Indonesia. The relations between allergic outcomes and infection and lifestyle factors will be addressed. This study was set up among school-age children in semi urban and rural areas, located in Ende District of Flores Island, Indonesia. A randomized placebo-controlled anthelmintic treatment trial to elucidate the impact of helminth infections on the prevalence of skin prick test (SPT) reactivity and symptoms of allergic diseases will be performed. The children living in these semi-urban and rural areas will be assessed for SPT to allergens before and after 1 and 2 years of treatment as the primary outcome of the study; the secondary outcome is symptoms (asthma and atopic dermatitis); while the tertiary outcome is immune responses (both antibody levels to allergens and cellular immune responses).

**Discussion:**

The study will provide information on the influence of helminth infections and anthelmintic treatment on immune response, atopy and allergic disorders.

**Trial registration:**

Current Controlled Trials ISRCTN: ISRCTN83830814

## Background

Helminth infections are highly prevalent worldwide, with more than two billion people chronically infected by soil transmitted helminths such as *Ascaris lumbricoides*, *Trichuris trichiura *and/or hookworms (*Necator americanus *or *Ancylostoma duodenale*) [1]. These enteric infections affect populations living in subtropical and tropical regions of low-middle income countries, where access to hygiene, sanitation and source of clear water is limited [2]. The immune responses mounted to helminth infections is characterized by T-helper type 2 (Th2), which are thought to be protective [3]. However, there is also evidence that these parasites might enhance their own survival by modulating the immune responses of their host by inducing regulatory responses that dampen activity of effector cells [4]. Whether all different helminths are equally potent in inducing regulatory responses is as yet not fully studied.

Allergens, like helminth antigens [5,6] are potent inducers of Th2 responses [7] and it is known that allergic diseases including asthma, eczema and rhinitis are associated with Th2 inflammation [8]. However, in contrast to helminth infections the Th2 associated allergic diseases, which are the most common cause of chronic disease of childhood in high income countries, appear to be less common in low income countries [9]. Thus, despite the close parallels between immune responses that characterize helminth infections and allergic diseases, namely increased levels of Immunoglobulin (Ig)-E, tissue eosinophilia and mastocytosis along with T cells that preferentially secrete Th2 cytokines interleukin (IL)-4, IL-5 and IL-13 [8,10-12], the clinical outcome with respect to immediate hypersensitivity and inflammation is clearly not the same [13]. Indeed, often it has been reported that these diseases, appear to segregate geographically [14] and several studies have reported a negative association between the presence of helminth infections and allergic disorders [15-18]. In experimental animal models, several parasitic helminths have been shown to prevent the development of eosinophilic airway inflammation and hyperresponsiveness [19-21].

Mechanistically, a number of immune responses have been proposed to account for the negative association between helminths and allergies [22]. The observations that chronic helminth infections are associated with higher suppressive responses, such as IL-10 [23] and regulatory T cells [24,25] have led to the proposal that a strong regulatory network induced by helminths might prevent the downstream effector phase of Th2 responses, preventing excess inflammation. Moreover, the possibility that in the presence of helminth infections, IgE antibodies generated are of lower affinity and therefore can not lead to mast cell degranulation has also been put forward [22].

Given that a number of studies have on the other hand reported either no [26,27] or a positive [28,29] association between helminths and allergies, it is very likely that apart from the source and chronicity of infection other factors such as exposure to non-helminth infections, and/or lifestyle play an important role in the development of allergies. The change from traditional to a more "modern" lifestyle which encompasses not only reduced exposure to micro-organisms and parasites but also an altered diet, in addition to changes in degree of manual labour or inhalation of pollutants is clearly associated with changing disease patterns [2]. It is important to study and delineate the mechanisms that may protect from the development of allergic diseases. It is becoming clear that the prevalence of allergic diseases is increasing in low to middle income countries [30] particularly in urban centers which often show higher prevalence of these diseases compared to rural areas [14,31,32]. It is therefore important to use this window of opportunity to identify risk and protective factors in cross sectional as well as longitudinal studies.

A study that would include both helminth infections and life style factors with respect to the development of allergies has been planned in Indonesia. The question of whether helminth infections play a role in allergic diseases can best be addressed by intervention studies. So far, one intervention study has suggested that anthelmintic treatment might increase the incidence of atopy reactivity [33], which is in contrast to a large scale study where one year after treatment of intestinal helminths no changes were recorded in allergic disorders [34]. None of the studies have been based on a large scale placebo controlled trial. Although there clearly are ethical issues with such a design, the ethics committee of University of Indonesia, has granted permission for a placebo controlled trial providing that the community gets extensive medical care and excludes those with intense infections. In addition to helminth infections, the study of how other factors may contribute to the development of allergies is best achieved by longitudinal comparison of different areas along a rural-urban gradient. Numerous studies have analyzed the difference in the prevalence of allergic disorders in a rural to urban gradient [35] but none so far has done so in a longitudinal manner with the exception of one study in Ghana [31].

The ImmunoSPIN allergy project http://www.immunospin.org[36] has been initiated with this aim. This study is a randomized placebo-controlled anthelmintic treatment trial to elucidate the impact of helminth infections on the prevalence of atopy and allergic diseases. In this study the prevalence of IgE, skin prick test positivity and symptoms of allergic diseases such as asthma and atopic dermatitis in school-age children will be assessed in semi-urban and rural area in Flores, Indonesia. The ImmunoSPIN allergy project will establish the risk and protective factors and will include immune response measurements in order to understand the immunological mechanisms that are behind risk and protective factors in allergy development.

## Methods/Design

### The study area

**Figure 1:**
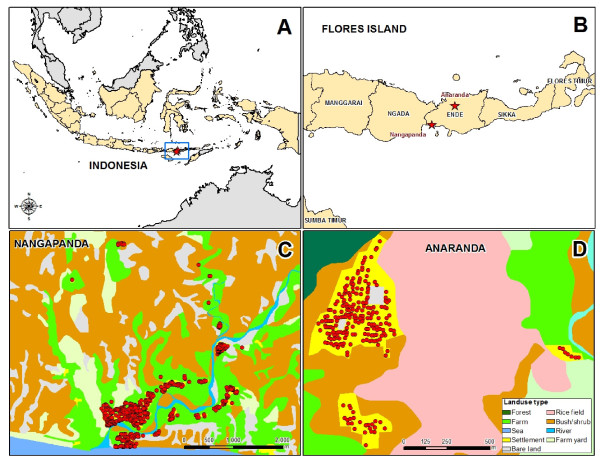
**The map of the study area in Flores, Indonesia**. A. Indonesia map. B. Study areas in Flores island which star sign. C. Nangapanda (semi urban area) where each dot represents one household. D. Anaranda (rural area) where each dot represents one household.

For this study semi-urban (Nangapanda) and rural (Anaranda) sites located in Ende District of Flores Island, Indonesia were selected. Nangapanda is a sub-district situated in a coastal area with a population of approximately 22,000 (Figure 1c.). Nangapanda is divided into 17 villages of which those located near the community health centre (Puskesmas), Ndeturea, Ndorurea1, and Ndorurea together with a population of 4650 [36] were included in the study. Local income in this area is based on fishing and farming while some engage in jobs at government officers with a few in the private sector.

Anaranda is a village in sub-district of Welamosa and is located 80 km north from Nangapanda with a population of approximately 1,600 (Figure 1d.). The majority of income is generated by farming. The infrastructure is poor with no paved roads which makes the village isolated and with little access to amenities as were available in Nangapanda, such as electricity (which is provided 12 hours per day), fuel, natural water source (not processed water) and shops.

Preliminary surveys in 2005 and 2006 found these areas to be endemic for geohelminths (*A. lumbricoides*, hookworms and *T. trichiuria*).

### Design

This study of schoolchildren is designed as a double-blind randomized trial with two arms. One arm is treatment with albendazole (single dose of 400 mg), while the other arm is treatment with matching placebo (both tablets from PT Indofarma Pharmaceutical, Bandung, Indonesia). The treatment will be provided every three months for a period of two years (total of 8 treatments). The resident population of the study area were randomized, by computer aided block randomization at household level, using Random Allocation Software [37] to either receive placebo or treatment. The treatment will be coded with random numbers and the code will be concealed from investigators and patients. Labels with the study subject ID will be printed from a computer database and attached to the appropriate strip of treatment by a separate team located in Jakarta without the involvement of the study investigators. Treatment codes will be unblinded by a monitoring committee after 1 year of treatment for interim analysis of any adverse effects that retention of anthelmintic treatment might have on the growth of children and on the incidence of allergy. If the trial continues, the final unblinding of the codes will take place after two years of treatment. At the end of the study the whole population will be treated.

From the total study population, schoolchildren aged 5-15 years will be included in the study of allergy parameters at pre, 1 and 2 years post treatment. The baseline demographic data as well as detailed questionnaires to delineate risk and protective factors for the development of allergies are planned at pre treatment stage. Skin prick testing (SPT) to allergens, International Study of Asthma and Allergy in Childhood (ISAAC) questionnaires, stool collection for helminth load, blood sampling for serology as well as whole blood culture is planned for time points pre, 1 and 2 years post treatment while exercise-induced bronchialconstriction (EIB) will be measured at pre and two years post treatment. Whereas SPT, ISAAC, parasitological examination as well as serology will be performed for all study subjects, where blood culture will be performed for children randomly selected based on households. The EIB will also be done in a subset of randomly selected children based on helminth as well as SPT status.

### Sample size

Data available from a pilot study where 102 schoolchildren were skin prick tested as well as data available from studies on other areas of Indonesia [38,39] were used for sample size calculations. Initial prevalence of SPT reactivity to a panel of allergens was found to be 15%. In order to find a 50% increase or decrease in SPT in the population, and taking into account a loss to follow-up of 20%, 709 individuals were needed in each treatment arm (taking into account a power of 0.90 and an alpha of 0.05). The reported prevalence of SPT to aeroallergens in children rural versus urban areas in low and middle income countries is around 10% versus 20% [31,40]. Moreover, treatment studies have often shown a doubling of SPT reactivity in treatment studies [4,33], we have based our sample size calculation on a more modest increase as the prevalence of helminth infections is high in Anaranda and Nangapanda areas and therefore it might be difficult to eliminate these infections.

### Information, recruitment, consent, specimen collection and storage

**Figure 2:**
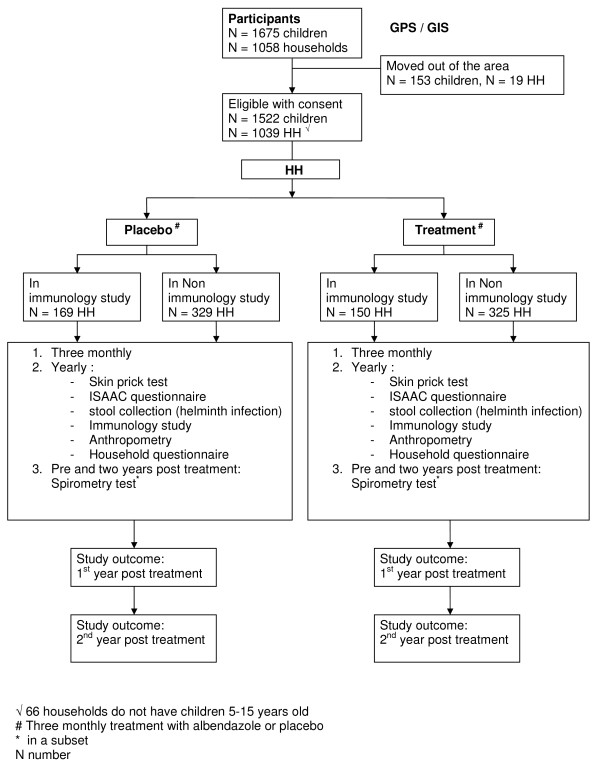
**Profile of the ImmunoSPIN-allergy sub project http://www.immunospin.org in study areas, Flores, Indonesia**.

The local health authorities in Ende were informed and they gave their agreement and support for this study. Socialization took place over a two-year period, from 2006 to 2008. Staff members from the Puskesmas are being fully involved and 50 community workers are being trained for following study subjects, filling questionnaires and keeping the community well-informed and well-engaged. Through many organized sessions, the village heads are being involved in passing on information about the study, including the benefits and risks involved. The longitudinal nature is explained and information sheets and consent forms (in Bahasa Indonesia) were distributed. Parents or guardians gave informed consent which was registered by signature or thumb print. Peripheral blood will be drawn once a year at baseline, 1 and 2 years after treatment for immunological studies. Stool samples will be collected once a year for intestinal helminth examination. Whole blood cultures will be set up using samples from individuals identified in a subset of households randomly selected from the treatment and placebo arms (Figure 2.). All blood samples (serum, cell pellet, plasma, and whole blood), blood culture supernatants, as well as stool samples for PCR, will be kept at -20°C and sent to Jakarta on dry ice to be kept at -20°C (plasma, cell plasma, blood culture supernatant) or -80°C (serum). At baseline, we mapped all houses using GPS system. All data that will be collected will be stored in an MS Access database (Microsoft, Redmond, WA, USA).

#### 1. Questionnaires and additional measurements

Additional factors that could influence allergy or atopy will be obtained by questionnaires. The questionnaires include the core allergy symptoms questions of ISAAC and information on history of disease and treatment in the last 12 months, treatment for worm infections, treatment for malaria infection, bednet use, material of blanket and mattress, habit to use shoes or sandals, exposure to smoking, family history of infectious diseases, BCG scar, history of immunization, history of breast feeding and food consumption. The questionnaires will be administered to the parent or guardian of each child under supervision of an interviewer. The same interviewer will dispense the ISAAC questionnaire during house to house visits. For core ISAAC questionnaires, we will show a video of asthma and rhinitis as well as pictures for dermatitis. Additional questionnaires will be held on socio-economic status, hygiene, ethnicity and environment factors. These questionnaires include information as material of the house, use of processed water, electricity, floor material, fuel, management of waste and exposure to pets and animals, and parent education level. Standing height and weight without shoes will be measured.

#### 2. Skin prick test

Skin prick test (SPT) reactivity to common aeroallergens [41] will be tested with extracts of *Dermatophagoides pteronyssinus *and *farinae *(HAL Allergy Laboratories, Leiden, The Netherlands) and *Blatella germanica *(Lofarma, Milan, Italy) and to four allergens with extracts of shrimp, soybean, peanut and fish (HAL Allergy). A histamine positive control and a saline negative control will be used to reduce false positives and negatives. SPT will be done on the volar side of the child's lower arm using skin prick lancets (Stallergènes SA, Antony, France). The wheal size will be measured after 15 minutes. Skin prick reactivity is be considered positive if the longest diameter of the wheal size plus the diameter perpendicular to it divided by two is at least 3 mm. All SPTs in the study will be performed by the same investigator.

#### 3. Spirometric and exercise challenge test

One hundred and twenty children selected on the basis of being helminth positive or negative and SPT positive or negative will be randomized to select equal number of males and females that fall into the four categories: helminth positive and SPT positive, helminth negative and SPT negative, helminth positive and SPT negative, helminth negative and SPT positive for spirometric test. This test will be performed before exercise, three minutes after exercise and at eight minutes after exercise. Each child will go through a vigorous six minutes free running exercise in the school playground and their heart rate will be recorded before and after exercise as a measurement of the level of exercise stress achieved. Spirometer calibrations will be done daily before use according to ambient atmospheric pressure and temperature. To determine whether EIB has occurred, we will compare the highest forced expiratory volume in one second FEV1 value from at least three acceptable trials before exercise with the lowest FEV1 from at least three acceptable trials after exercise. Spirometric tests will be performed using a portable spirometer (Jaeger, Germany) with the child sitting and with a nose clip. EIB will be defined as positive if FEV1 falls by 15% or more after exercise.

#### 4. Parasitological examination

##### Stool examination by microscopy

The formol-ether acetate concentration method [42] will be performed on the formalin preserved stool samples followed by microscopic examination for intestinal helminth infections, as well as protozoan infections. For the detection of hookworm larvae, an amount of fresh stool sample will be cultured using filter paper soaked by distilled water inside a sealed plastic bag according to the Harada Mori method and the presence of larvae will be determined by microscopic examination after seven days [43].

##### Stool examination by real-time PCR

Stool examination by real time PCR will be done in the Netherlands as previously described [36]. Briefly, DNA will be extracted from unpreserved stool samples were stored at -20°C). A multiplex real-time PCR will be used for the specific amplification and detection of *A. duodenale-, N. americanus-, A. lumbricoides*, and *S. stercoralis *DNA [44,45]. Amplification reactions will be performed in white PCR plates in a volume of 25 μl with PCR buffer (Hotstar Taq master mix, QIAgen, Germany), 5 mM MgCl2, 2.5 μgram Bovine Serum Albumin (Roche Diagnostics Nederland B.V., Almere, the Netherlands), 5 pmol of each *A. duodenale*-specific primer, and of each *N. americanus*-specific primer, 2 pmol of each *A. Lumbricoides*-specific primer, 2.5 pmol of each *S. stercoralis*-specific primer and 3.75 pmol of each PhHV-1-specific primer, 1.25 pmol of each *N. americanus*-specific XS-probe, *A. lumbricoides*-specific XS-probe, *S. stercoralis*-specific double-labelled probe, and PhHV-1-specific double-labelled probe, and 2.5 pmol of the *A. duodenale*-specific XS-probe, and 5 μl of the DNA sample.

Amplification consists of 15 min at 95°C followed by 50 cycles of 15 s at 95°C, 30 s at 60°C, and 30 s at 72°C. Amplification, detection, and analysis will be performed with the CFX real-time detection system (Bio-Rad laboratories). The PCR output from this system consists of a cycle-threshold (Ct) value, representing the amplification cycle in which the level of fluorescent signal exceeds the background fluorescence reflecting the parasite-specific DNA load in the sample tested. Negative and positive control samples are included in each amplification run.

The amplification is considered to be hampered by faecal inhibitory factors if the expected cycle threshold (Ct) value of 33 in the PhHV-specific PCR is increased by more than 3.3 cycles.

#### 5. Whole blood culture and cytokine measurements in supernatants

Whole blood culture will be undertaken in the field studies and cytokine measurements will be done in Jakarta, Indonesia as described previously [36], Briefly, the heparinized blood will be diluted 1:4 with RPMI 1640 medium (Invitrogen, Breda, The Netherlands) and cultured in 96 well round bottomed plates. Stimulations will be performed with medium/control, PHA (2 μg/ml, Wellcome Diagnostics, Darford, UK), LPS (1 ng/ml Sigma-Aldrich, Zwijndrecht, The Netherlands), Pam3Cys (100 ng/ml, Cayla-InvivoGen Europe, Toulouse, France), PolyIC (50 μg/ml, Cayla-InvivoGen Europe, Toulouse, France) and *Ascaris *antigen (20 μg/ml as prepared by van Riet E et al [46]. Supernatants will be collected on day 1 (unstimulated control, LPS, Pam3Cys) and day 3 (unstimulated control, PHA, Ascaris, PolyIC). TNF-α and IL-10 from day 1 supernatants as well as IL-2, IL-5, IL-10, IFN-γ, and TNF-α for day 3 supernatants will be analysed simultaneously using commercial Luminex cytokine kit (Biosource, Camarillo, CA, USA) and run on a Liquichip 200^® ^Workstation (Qiagen, Venlo, The Netherlands) equipped with Liquichip analyzer software (Qiagen, Venlo, The Netherlands).

#### 6. Antibody measurements

##### Antibody IgE measurement

Measurement of plasma specific IgE to *Ascaris lumbricoides *antigen and to *Dermatophagoides pteronyssinus *(house dust mite), *Blatella germanica*, shrimp and peanut will be performed using an ImmunoCAP 250 system (Phadia, Uppsala, Sweden) following the manufacturer's instructions [47]. All measurement will take place in one laboratory in the Netherlands.

##### Total IgE

Total IgE will be measured in Jakarta, Indonesia as described previously[36,48]. Briefly, maxisorp plates (Thermo Fisher Scientific, Roskilde, Denmark) will be coated overnight with 1/1400 diluted rabbit anti-human IgE (Dako, Glostrup, Denmark). Plates are blocked with phosphate buffered saline (PBS) containing 5% bovine serum albumin (BSA, Albumin Fraction V, Boehringer, Mannheim, Germany). Sera is diluted 1/200 in PBS containing 5% fetal calf serum (FCS, Greiner Bio-One, Alphen a/d Rijn, Netherlands). A positive standard serum containing human IgE (NIBSC, Potters Bar, UK) are incubated on each plate. Plates will be incubated for 1 hour at room temperature. After a washing step, IgE biotinylated goat anti-human IgE antibody (1/1000 (Vector Laboratories, Burlingame, CA, USA)) and Streptavidin Alkaline Phosphatase conjugate (1/3000 (Boehringer, Mannheim, Germany)) will be incubated for 1 hour at room temperature. The results will be expressed in International Units (IU).

### Outcomes and case definitions

The study aims to determine whether and how helminth infections may affect allergic disorders. The study will therefore determine the effect that anthelmintic treatment albendazole has versus placebo on SPT reactivity, symptoms of allergic diseases and immune responses. The primary outcome of the study is SPT; the secondary outcome is symptoms (asthma and atopic dermatitis); while the tertiary outcome is immune responses (both IgE levels to allergens and cellular immune responses that represent both innate and adaptive immune reactivities).

Helminth infections will be determined by the presence of parasites detected by microscopic examination of stool as well as by molecular (PCR-based) methods. Atopy will be defined as either positive in SPT (≥3 mm wheal size) to any of aeroallergens tested or the presence of allergen-specific IgE ≥ 0.35 kU/L. Symptoms of asthma and atopic dermatitis will be assessed by modified ISAAC questionnaires [41] that have been translated into Bahasa Indonesia and translated back to English and adapted for use in the study area. It has been tested in some areas in Sulawesi as well as in study areas during pilot studies. A positive answer to the questions (i) has your child ever had asthma? (ii) has this asthma been diagnosed by a doctor? and (ii) has your child ever or in the past 12 months had wheezing or whistling in the chest? will be interpreted as asthma, while a positive answer to the question (iv) has your child ever had doctor/paramedic diagnosed allergic eczema and (v) has your child ever had one or more skin problem accompanying an itchy rash? will be taken as atopic dermatitis.

In order to test the effect of helminths on bronchial hyper-responsiveness (BHR) we will test lung function following American Thoracic Society and European Respiratory Society (ATS/ERS) guidelines[49] for lung function and BHR testing [41,50,51]. FEV1 will be measured before and after 6 minutes of exercise [52] and a fall of 15% in FEV1 is considered to indicate EIB. In order to take into account confounding factors, data on family structure like number of siblings, birth order will be recorded as well as details of birth and breastfeeding, hygiene, socioeconomic status, annual health status, and food consumption.

### Overview of plan of analyses

The baseline data will be analyzed to determine whether helminth infections are associated with allergen specific IgE, atopy and symptoms of asthma or eczema. Both presence of infection and intensity of infection will be used for logistic and linear regression analyses. Analysis will be adjusted for confounding factors such as socio-economic status, body mass index, age, sex and environmental exposure. Additional confounders that are identified during the study will also be used. In order to examine a general effect of parasite burden and rural and semi-urban differences in atopy and allergy will be compared. The effect of anthelmintic treatment on atopy and allergy will be assessed 1 and 2 years after treatment by analyzing prevalence as well as incidence. The analyses will be based on intention to treat approach. The groups in treatment and placebo arms will be compared as well as groups in whom helminth infection was reduced or remained unaltered irrespective of treatment assignment and we will also look at chronic versus acute infections, based on continuous presence of infection or newly gained infections over the follow up period. Individuals that are lost to follow up and individuals that are analyzed will be compared on the basis of their baseline characteristics, age, gender, village, socioeconomic status and parasitic infections. A similar comparison will also be undertaken to compare the characteristics of individuals in the treatment and placebo groups at inclusion into the study. *Chi*-square, t-tests and Mann-Whitney tests will be used to test for differences. For data-analyses we will use SPSS (SPSS Inc., Chicago, IL, USA) and ArcGis (ESRI; Redlands, CA, USA).

To summarize, the plan is to measure the prevalence of allergy in school-aged children in semi urban and rural areas, and to establish their association with helminth infections as well as various risk and protective factors. By studying cellular immunological parameters, it is also the aim to understand the immunological mechanisms that are behind risk and protective factors in allergy.

**Figure 3:**
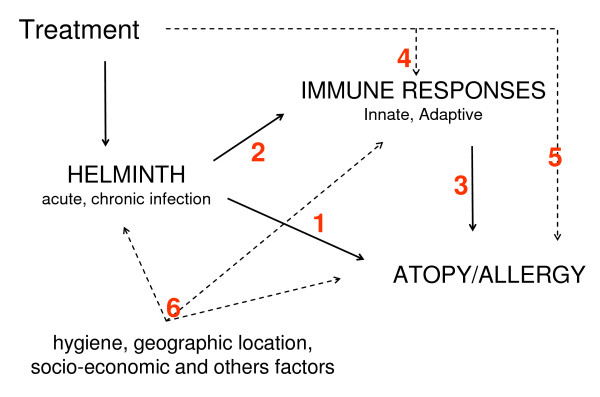
**Conceptual of principal question study**.

The analysis will be divided into six principals study questions (Figure 3.):

1. What is the association between helminth infections and atopy and allergy symptoms?

2. What is the association of helminth infections and immune responses?

3. What is the association between immune responses and atopy and allergy symptoms?

4. Does the immune response change after anthelmintic treatment?

5. Does the prevalence of atopy and allergy symptoms change after anthelmintic treatment?

6. What is the role of hygiene, geographic location and socio-economic status in helminth infection and allergy?

### Ethical consideration and trial registration

The study was approved by the Ethical Committee of the Medical Faculty, University of Indonesia, Jakarta (ethical clearance ref: 194/PT02.FK/Etik/2006) and has been filed by ethics committee of the Leiden University Medical Center, The Netherlands. The trial was registered as clinical trial ref: ISRCTN83830814. Parental consent was obtained for children who participated in the study. The study is reported in accordance with the CONSORT guidelines for cluster-randomized studies.

## Discussion

The prevalences of atopic disorders and asthma have been reported to be lower in low income than in high income countries, however these are becoming an increasingly important public health problem, particularly in urban centres of the developing world [35,53]. With the view to the increasing urbanization it is important to have data on factors and mechanisms underlying the development of allergic diseases in low to middle income countries. Indonesia is a prototype of a country in transition towards a well developed economy with dynamic changes in lifestyle. The ImmunoSPIN project was designed to assess how helminth infections can influence the immune response and clinical outcome of allergic disease and this study will compare atopy and allergic symptoms in children living in a semi urban and rural setting in Indonesia. The relations between allergic outcomes and the numerous measured exposures will be addressed.

In our study design we will use a longitudinal approach to assess the effect of anthelmintic treatment on prevalence, risk and protective factors in children living in semi urban and rural environments. The laboratory component will explore the relative importance of immunological mechanisms that are leading to increase in prevalence of allergic disorders. Statistical analysis will involve the use of strategies that link data from different levels (e.g. socio-economic, environmental, clinical and immunological factors) and will use advanced statistical techniques to deal with this complex mechanism.

**Figure 4:**
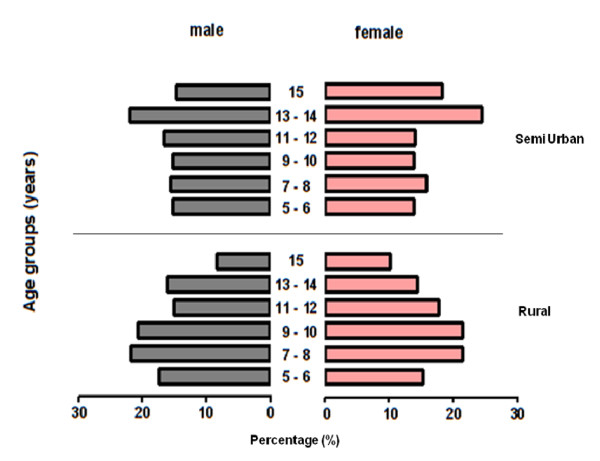
**Distribution of sex and age in school children participating in the study**. Distribution of children participants in the semi urban (Nangapanda) and the rural (Anaranda) areas.

The study has so far provided data that are shown in a flow chart given in Figure 2. A total of 1675 children were registered in 1058 households in study area, characteristics of study population are given in table 1 with prevalence of helminth infection in both areas. Distribution of sex and age groups are shown in (Figure 4.) in the semi urban area which has three junior high schools and four elementary schools, as well as in the rural area only one elementary school. This results in larger number of older children in semi urban area than in rural area. Some of the children exits from rural area leave the area to go to junior high school in surrounding larger villages but not necessarily to our semi urban study area (as the distance between our study areas is considerable). These children will be skin prick tested, and characterized via questionnaires. In addition, blood withdrawal will allow the determination of total and specific IgE in addition to cellular immunological measurements and in a sub sample the spirometric test will be performed. After completion of the study the whole population will be treated with adequate treatment for helminth infections. This study is also unique in that it will provide data on anthelmintic treatment efficacy and effectiveness in a defined large population in a developing country.

**Table 1:** Characteristics of the study population

	Semi Urban	Rural
Participants	1161	514
Age (mean, SD)	6.77 ± 3.31	5.85 ± 3.12
Sex male/female (male%)	583/578 (50.2)	270/244 (52.5)
BMI (mean, SD)	15.4 ± 2.39	14.4 ± 1.83
Parasites (%)		
Any helminths	53.2	40.8
*Ascaris lumbricoides*	38.7	21.8
*Trichuris trichiura*	25.3	11.8
Hookworms	11.8	18.5

Body Mass Index (BMI) = Weight(kg)/height(m) squared. Helminth infection was measured by microscopic examination.

If helminth infections are proved to be associated with reduced allergic disorders, and the mechanisms behind such protective effect is elucidated, measures can be taken to ensure that the vicious circle of westernization and increased allergy is prevented; for example vaccination with microbial products and allergens. If helminths are proved not to be associated but new factor(s) are identified along with the immunological mechanism(s) through which the development of allergies is affected, then appropriate preventive measures can also be planed.

In summary the ImmunoSPIN helminth-allergy study is the first and currently the only longitudinal study of helminth and allergy in Indonesia. The study has received enthusiastic support from the authorities in Ende and at the regional level. At the same time, the study facilitates the transfer of state of the art technologies in immunology, molecular biology, epidemiology and statistics to Indonesia.

## Competing interests

The authors declare that they have no competing interests.

## Authors' contributions

FH is medical doctor in charge of the field study involved in setting up the study, supervising gathering of data, clinical care, follow up of the study population and wrote the paper study; AEW is medical doctor in charge of the field study involved in setting up the study, supervising gathering of data, clinical care, and follow up of the study population; LJW is medical doctor in charge of the field study involved in setting up the study, supervising gathering of data, clinical care, and follow up of the study population; MMMK is biologist who is involved in the microscopic and molecular diagnosis of parasitic infections, BL is medical doctor who is the advisor on databases as well as epidemiological and statistical aspects of the study, IA is medical doctor who is the advisor on databases as well as epidemiological and statistical aspects of the study, HWU is statistician who is developing methods to analyze the complex data generated during the lifetime of the project, HW is parasitologist and field study expert who is in charge of the process of data selection, storage, safeguarding randomization, and privacy of the study subjects; YD is medical doctor who advises on the immunological aspects of the study, SW is medical doctor who is supervising the study set up, in particular assessment of clinical allergy, RS is lung function expert who is involved in analyzing spirometry results, JJV is molecular parasitologist who is involved in the molecular diagnosis of parasitic infections, RvR is immunologist who specializes in immunology of allergic disease, LM is immunoepidemiologist who is advising on databases maintenance, epidemiological, statistical, immunological aspects of the study and supervised the writing of the manuscript; ES is immunoparasitologist who is involved in coordinating the study and advising on parasitological and immunological aspects of the study, MY is immunologist who has developed the study, supervised the writing of the manuscript, and is the Dutch coordinator of the ImmunoSPIN program; TS is parasitologist who has developed the study and is the Indonesian coordinator of the ImmunoSPIN program. All authors read and approved the final manuscript.

## Pre-publication history

The pre-publication history for this paper can be accessed here:

http://www.biomedcentral.com/1471-2334/11/83/prepub
